# Prevalence of Malnutrition Among School‐Aged Children With and Without Intellectual and Developmental Disabilities in Saudi Arabia: A Cross‐Sectional Study

**DOI:** 10.1002/fsn3.71816

**Published:** 2026-04-25

**Authors:** Lujain A. Almousa, Naseem M. Alshwaiyat, Jozaa Z. ALTamimi, Reham I. Alagal, Malak A. Alsemari, Nora A. AlFaris

**Affiliations:** ^1^ Department of Health Sciences, College of Health and Rehabilitation Sciences Princess Nourah bint Abdulrahman University Riyadh Saudi Arabia; ^2^ Department of Nutrition and Food Technology, Faculty of Agriculture Jordan University of Science and Technology Irbid Jordan; ^3^ Department of Sports Health, College of Sports Sciences & Physical Activity Princess Nourah bint Abdulrahman University Riyadh Saudi Arabia; ^4^ Department of Medical Imaging–MRI, King Abdullah bin Abdulaziz University Hospital (KAAUH) Princess Nourah bint Abdulrahman University Riyadh Saudi Arabia

**Keywords:** intellectual and developmental disabilities, malnutrition, Saudi Arabia, school‐aged children

## Abstract

Globally, millions of children live with intellectual and developmental disabilities (IDD), which can raise the risk of malnutrition in children. Data from Saudi Arabia on school‐aged children with IDD remain limited. Therefore, this study aimed to determine the prevalence of malnutrition among school‐aged children with and without IDD in Saudi Arabia. This cross‐sectional study was conducted during August–December of 2024 at the Child Development Center at University Hospital, Riyadh, Saudi Arabia. School‐aged children (5–17 years) with clinician‐diagnosed autism spectrum disorder (ASD), Down syndrome (DS), attention deficit/hyperactivity disorder (ADHD), or cerebral palsy (CP) and age‐matched children without IDD were recruited. Weight and height were measured, and body mass index (BMI) was calculated. WHO Growth Reference 2007 (5–19 years) was used to generate BMI‐for‐age z‐scores (BAZ) and height‐for‐age z‐scores (HAZ) using WHO AnthroPlus; weight‐for‐age z‐scores (WAZ) were computed only for children aged ≤ 10 years. Malnutrition was defined as undernutrition (thinness and/or stunting and/or underweight) and/or overnutrition (overweight and/or obesity). A total of 168 children participated (IDD, *n* = 68; non‐IDD, *n* = 100). Overall malnutrition prevalence was similar in children with and without IDD (47% vs. 48%; risk ratio [RR] 0.98, 95% CI 0.71–1.36; *p* = 0.905). Overnutrition was more common than undernutrition in both groups (IDD: 29% overnutrition vs. 25% undernutrition; non‐IDD: 32% vs. 28%). Although overall prevalence was comparable, marked heterogeneity was observed across IDD subtypes (malnutrition: DS 90%, ADHD 50%, CP 48%, ASD 24%; *χ* ^2^ (3) = 12.02, *p* = 0.007; FDR‐adjusted *q* = 0.022). Concurrent stunting and overweight/obesity (individual‐level double burden) was observed in 12% of non‐IDD children and 7% of children with IDD. In conclusion, nearly half of this hospital‐based sample of Saudi school‐aged children had evidence of malnutrition, with overnutrition exceeding undernutrition. While overall malnutrition prevalence did not differ between children with and without IDD, the distribution across IDD subtypes was clinically meaningful. Routine growth monitoring and targeted nutrition support are needed in both clinical and school settings.

## Graphical abstract

Malnutrition among Saudi school‐aged children with and without intellectual and developmental disabilities (IDD). ADHD, attention‐deficit/hyperactivity disorder; ASD, autism spectrum disorder; CP, cerebral palsy; DS, Down syndrome; IDD, intellectual and developmental disabilities; Non‐IDD, children without intellectual and developmental disabilities.

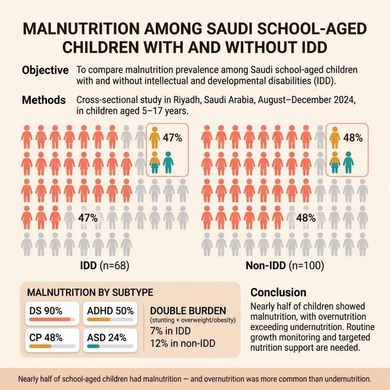

## 1 Introduction

Malnutrition, defined as an inadequate or imbalanced intake of energy and/or essential nutrients, includes both undernutrition (e.g., stunting, thinness, underweight) and overnutrition (overweight and obesity). Unfortunately, malnutrition can interrupt normal child growth and development, which may have lifelong negative effects (De and Chattopadhyay 2019). It remains a major global public health challenge and is shaped by multi‐level determinants including household income, care practices, education, and the food environment (Katoch 2022, 2024).

Intellectual and developmental disabilities (IDD) are conditions that start in childhood and affect cognition, behavior, communication, or motor function (WHO 2024). These neurodevelopmental disorders include autism spectrum disorder (ASD), Down syndrome (DS), attention‐deficit/hyperactivity disorder (ADHD), and motor disorders such as cerebral palsy (CP) (WHO 2024). Children with IDD experience unique health risks that negatively affect their nutritional status. For example, sensory dysfunction, frequently seen in ASD, may limit dietary choices; hypotonia, endocrine comorbidities, and lower resting energy expenditure, typically observed in DS, may increase weight gain risk; stimulant medication and behavioral dysregulation, often seen in ADHD, can impair appetite and regular meals; and oral‐motor dysfunction and dysphagia, commonly observed in CP, can reduce food intake (Sharp et al. 2013; Basil et al. 2016; Zhu et al. 2024; Rebelo et al. 2022). These health challenges can result in a double burden of malnutrition, manifesting as increased risks of both undernutrition, such as wasting, stunting, and micronutrient deficiencies, and overnutrition, such as overweight and obesity, compared to children without IDD (Viana et al. 2025; Yan et al. 2025; Mohamed et al. 2021).

High rates of feeding problems have been reported in ASD and ADHD, obesity has been reported in DS, and prevalent undernutrition has been reported in CP, highlighting the need for routine nutritional assessment and individualized dietetic care for children with IDD (Sharp et al. 2013; Bertapelli et al. 2016; Cortese et al. 2016; da Silva et al. 2022). Systematic reviews have consistently shown increased risk of both obesity and underweight in children with ASD, a pattern usually caused by selective eating, sleep and activity variations, and side effects of medications (Sharp et al. 2013; Kahathuduwa et al. 2019, 2022). A comprehensive review found overweight and obesity to be more frequent in children with DS than in the general pediatric population, with combined prevalence rates ranging from 23% to 70% across studies, attributed to multifactorial determinants including reduced resting energy expenditure, hypothyroidism, and low physical activity (Bertapelli et al. 2016). In addition, a systematic review found a small but substantial association with obesity in children with ADHD, likely caused by impulsivity, sleep, and activity patterns. Furthermore, appetite‐suppressing medications can transiently lower weight gain, emphasizing the importance of growth monitoring (Cortese et al. 2016). A systematic review reported a pooled prevalence of malnutrition of about 40% in children with CP, attributed to factors including feeding difficulties, dysphagia, gastrointestinal morbidity, and mobility constraints (da Silva et al. 2022). Overall, many children with IDD are at higher risk of both undernutrition and overnutrition than typically developing children.

In fact, many countries around the world, including those in the Middle East, face a double burden of malnutrition in which high rates of overweight and obesity coexist with persistent stunting and thinness, particularly among children and adolescents. A pooled analysis of worldwide trends showed that obesity prevalence has risen and now exceeds thinness in many settings among 5–19‐year‐olds (NCD‐RisC 2024), and the individual‐level double burden of malnutrition has been increasingly documented (Viana et al. 2025).

Saudi Arabia is experiencing a nutritional transition with high levels of childhood overweight and obesity, while stunting and other forms of undernutrition persist in some subgroups. Regional estimates suggest around one‐third of school‐aged children and adolescents in the Middle East and North Africa (MENA) are overweight or obese, with Saudi Arabia among the countries with particularly high prevalence (UNICEF MENA 2024). School environments are a strategic venue for prevention; however, available evidence suggests only moderate compliance with school canteen nutrition policies in Riyadh and continued availability of energy‐dense foods (Aldubayan and Murimi 2019).

Although international literature suggests higher malnutrition risk in children with IDD, Saudi data on school‐aged children, particularly comparing children with and without IDD using standardized WHO growth references, remain scarce (AlFaris et al. 2024). Moreover, subgroup heterogeneity across IDD conditions may be clinically important but is often masked by aggregate analyses. Therefore, this study aimed to estimate the prevalence of malnutrition among Saudi school‐aged children with and without IDD and to describe differences across IDD subtypes.

## 2 Methods

### 2.1 Study Design and Participants

This cross‐sectional study was conducted in Riyadh, Saudi Arabia, from August to December 2024. Eligible participants were Saudi children aged 5–17 years living in Riyadh. The IDD group comprised children with clinician‐diagnosed ASD, DS, ADHD, or CP, as documented in electronic medical records. Children were recruited consecutively (convenience consecutive sampling) as they attended the Child Development Center, University Hospital, Riyadh, Saudi Arabia, during the study period. The comparison group comprised children without IDD recruited from general pediatric outpatient clinics at the same hospital during the same period. Children in the comparison group had no documented neurodevelopmental disorder and no parent‐reported disability. Where feasible, comparison participants were frequency‐matched to the IDD group by age and sex. The hospital‐attending comparison group was used for feasibility and to reduce measurement differences (same setting and anthropometric protocol), while emphasizing that this may overestimate malnutrition prevalence in the non‐IDD group and attenuate group differences. Children referred to the hospital with their parents were invited to participate in this study after obtaining the parents' approval in the form of written consent according to the Helsinki Declaration. The study protocol was approved by the research ethics committee of Princess Nourah bint Abdulrahman University, Riyadh, Saudi Arabia.

An a priori sample size was estimated for the primary comparison of overall malnutrition prevalence between children with and without IDD. Assuming a malnutrition prevalence of 35% in children without IDD and 55% in children with IDD (20 percentage‐point absolute difference), a two‐sided *α* = 0.05 and 80% power would require approximately 96 participants per group. Because recruitment was clinic‐based and time‐limited, the achieved sample (*n* = 100 without IDD; *n* = 68 with IDD) was lower than the target for the IDD group; therefore, analyses, especially IDD subtype comparisons, should be interpreted as exploratory, and the possibility of Type II error is acknowledged.

### 2.2 Data Collection

Electronic medical records and personal interviews with children's parents were used to collect data about the types of disability and sociodemographic information. Data about the type and severity of the disability were collected from the medical portfolios of children based on medical assessments performed by clinicians. The targeted participants were children with one of four types of IDD: ASD, DS, ADHD, or CP, as well as age‐matched healthy children. Weight and height were measured to assess the level of malnutrition according to WHO standards for child growth assessment. Weight was measured using a standard digital weight scale, and height was measured using a stadiometer in the standing position, with measurements recorded to the nearest 0.1 kg and 0.1 cm, respectively. Body mass index (BMI) was calculated by dividing weight in kilograms by the square of height in meters.

### 2.3 Malnutrition Assessment

WHO Growth Reference 2007 for children and adolescents aged 5–19 years was used to compute sex‐ and age‐standardized z‐scores in WHO AnthroPlus (De Onis et al. 2007; WHO 2009). Thinness was defined as BMI‐for‐age z‐scores (BAZ) < −2 SD and severe thinness as BAZ < −3 SD. Overweight was defined as BAZ > +1 SD and ≤ +2 SD, and obesity as BAZ > +2 SD. Stunting was defined as height‐for‐age z‐scores (HAZ) < −2 SD and severe stunting as HAZ < −3 SD. Underweight (weight‐for‐age z‐scores (WAZ) < −2 SD; severe underweight WAZ < −3 SD) was assessed only for children aged ≤ 10 years, consistent with WHO recommendations (De Onis et al. 2007). Undernutrition was defined as thinness and/or stunting and/or underweight (where applicable), and overnutrition as overweight and/or obesity. Malnutrition was defined as the presence of undernutrition and/or overnutrition.

### 2.4 Statistical Analysis

Analyses were performed using SPSS (version 26). Categorical variables were summarized as counts and percentages. Group comparisons (IDD vs. non‐IDD) were conducted using the chi‐squared test. To support interpretability, we report chi‐square statistics, degrees of freedom, and *p*‐values, and we estimate risk ratios (RR) with 95% confidence intervals (CI) for primary outcomes. For comparisons across IDD subtypes (ASD, DS, ADHD, CP), chi‐squared tests were used, and effect sizes were summarized using Cramer's *V*. Because multiple subtype comparisons were performed, false discovery rate (FDR) adjusted *q*‐values (Benjamini–Hochberg) are also reported for subtype analyses. Two‐tailed *p* < 0.05 were considered statistically significant.

## 3 Results

In total, 168 school‐aged children participated in this study (68 with IDD and 100 without IDD). The IDD children had ASD (*n* = 21), DS (*n* = 10), ADHD (*n* = 16), or CP (*n* = 21). Table 1 presents sociodemographic characteristics of study participants stratified by disability status and type. About half of the study participants, either with or without IDD, were males and aged 5–10 years.

**TABLE 1:** Sociodemographic characteristics of study participants stratified by disability status.

Variable^a^	Healthy children (*n* = 100)	Disabled children (*n* = 68)	*p*	ASD (*n* = 21)	DS (*n* = 10)	ADHD (*n* = 16)	CP (*n* = 21)	*p*
Age (years)								0.991
5–10	48 (48%)	36 (53%)	0.53	11 (52%)	5 (50%)	9 (56%)	11 (52%)	
11–17	52 (52%)	32 (47%)		10 (48%)	5 (50%)	7 (44%)	10 (48%)	
Gender								
Males	48 (48%)	39 (57%)	0.234	14 (67%)	5 (50%)	9 (56%)	11 (52%)	0.756
Females	52 (52%)	29 (43%)		7 (33%)	5 (50%)	7 (44%)	10 (48%)	
Family income								
Less than 10,000 SR	46 (46%)	41 (60%)	0.069	8 (38%)	6 (60%)	13 (81%)	14 (67%)	0.055
10,000 SR or more	54 (54%)	27 (40%)		13 (62%)	4 (40%)	3 (19%)	7 (33%)	
Home care provider								
Parents	74 (74%)	50 (74%)	0.218	15 (71%)	4 (40%)	16 (100%)	15 (71%)	**0.026**
Mother	17 (17%)	17 (25%)		5 (24%)	6 (60%)	0 (0%)	6 (29%)	
Father	2 (2%)	0 (0%)		0 (0%)	0 (0%)	0 (0%)	0 (0%)	
Grandparents	7 (7%)	1 (2%)		1 (5%)	0 (0%)	0 (0%)	0 (0%)	

^a^ Categorical variables were analyzed by using chi‐squared test and expressed as numbers and percentages. Significant values (*p* < 0.05) were presented in bold type.

Table 2 presents the prevalence of malnutrition among study participants stratified by disability status. Overall malnutrition prevalence, defined as undernutrition and/or overnutrition, did not differ between children with and without IDD (47% vs. 48%; *χ* ^2^(1) = 0.01, *p* = 0.905; RR = 0.98, 95% CI = 0.71–1.36). Similarly, undernutrition (25% vs. 28%; *χ* ^2^(1) = 0.19, *p* = 0.666; RR = 0.89, 95% CI = 0.53–1.50) and overnutrition (29% vs. 32%; *χ* ^2^(1) = 0.13, *p* = 0.722; RR = 0.92, 95% CI = 0.58–1.46) were comparable between groups. These null findings should be interpreted cautiously, given the hospital‐based comparison group and modest sample size, which may limit power to detect differences.

**TABLE 2:** Prevalence of malnutrition indicators in study participants with and without IDD.

Variables^a^	Children without IDD (*n* = 100)	Children with IDD (*n* = 68)	*χ* ^2^ (df)	*p*	RR (95% CI)
Underweight^b^					
Yes	3 (6%)	5 (13%)	1.42 (1)	0.233	2.25 (0.57–8.87)
No	51 (94%)	35 (88%)			
Severe underweight^b^					
Yes	1 (2%)	2 (5%)	0.74 (1)	0.391	2.70 (0.25–28.75)
No	53 (98%)	38 (95%)			
Stunting					
Yes	19 (19%)	12 (18%)	0.05 (1)	0.824	0.93 (0.48–1.79)
No	81 (81%)	56 (82%)			
Severe stunting					
Yes	13 (13%)	6 (9%)	0.70 (1)	0.401	0.68 (0.27–1.70)
No	87 (87%)	62 (88%)			
Thinness					
Yes	9 (9%)	7 (10%)	0.08 (1)	0.779	1.14 (0.45–2.92)
No	91 (91%)	61 (90%)			
Severe thinness					
Yes	6 (6%)	4 (6%)	0.00 (1)	0.975	0.98 (0.29–3.34)
No	94 (94%)	64 (94%)			
Undernutrition					
Yes	28 (28%)	17 (25%)	0.19 (1)	0.666	0.89 (0.53–1.50)
No	72 (72%)	51 (75%)			
Severe undernutrition					
Yes	19 (19%)	9 (13%)	0.97 (1)	0.325	0.70 (0.34–1.45)
No	81 (81%)	59 (87%)			
Overweight					
Yes	20 (20%)	10 (15%)	0.77 (1)	0.379	0.74 (0.37–1.47)
No	80 (80%)	58 (85%)			
Obese					
Yes	12 (12%)	10 (15%)	0.26 (1)	0.61	1.23 (0.56–2.68)
No	88 (88%)	58 (85%)			
Overnutrition					
Yes	32 (32%)	20 (29%)	0.13 (1)	0.722	0.92 (0.58–1.46)
No	68 (68%)	48 (71%)			
Malnutrition					
Yes	48 (48%)	32 (47%)	0.01 (1)	0.905	0.98 (0.71–1.36)
No	52 (52%)	36 (53%)			

^a^ Categorical variables were analyzed by using chi‐squared test and expressed as numbers and percentages. Significant values (*p* < 0.05) were presented in bold type.

^b^ All participants (≤ 10 years); *n* = 94 (children with IDD; *n* = 40).

Specifically, underweight was found in only 6% of healthy participants and 13% of disabled participants, while severe underweight was found only in one healthy child (2%) and two disabled children (5%) (Table 2). Stunting was shown in 19% of healthy participants and 18% of disabled participants, while severe stunting was shown in 13% of healthy participants and 9% of disabled participants. Moreover, thinness was reported in 9% of healthy children and 10% of children with IDD, while 6% of healthy children and 6% of disabled children were severely thin. On the other hand, the prevalence of overweight among healthy and disabled children was 20% and 15%, respectively, while 12% of healthy participants and 15% of disabled participants were obese. Remarkably, there were no significant differences in the prevalence of these forms of malnutrition between children with and without IDD.

The prevalence of malnutrition, defined as undernutrition and/or overnutrition, among study participants with IDD stratified by disability type is presented in Table 3. Overall, malnutrition prevalence was significantly higher among children with DS (90%) than among children with ADHD (50%), CP (48%), or ASD (24%) (*p* = 0.007). The prevalence of undernutrition was significantly higher among participants with DS (60%) or ADHD (50%) than those with CP (14%) (*p* = 0.001). Undernutrition was not reported among autistic children. However, severe undernutrition was observed only among children with DS (40%) or ADHD (31%). Among children with IDD, underweight was only reported among those with ADHD (30%) or CP (17%), while severe underweight was reported only in two disabled children diagnosed with ADHD (20%). Interestingly, stunting was significantly higher among participants with DS (50%) than those with ADHD (38%) or CP (5%) (*p* = 0.001). Similarly, 20% of children with DS and 25% of children with ADHD were severely stunted. Furthermore, 20%, 19%, and 10% of children with DS, ADHD, and CP were thin, respectively, and 20% of children with DS and 13% of children with ADHD were severely thin. On the other hand, overnutrition (either overweight or obese) was reported among 24%, 50%, 19%, and 33% of children with ASD, DS, ADHD, and CP, respectively (Table 3). Among participants with IDD, 10%, 10%, 13%, and 24% of children with ASD, DS, ADHD, and CP were respectively overweight, while 14%, 40%, 6%, and 10% of children with ASD, DS, ADHD, and CP were respectively obese.

**TABLE 3:** Prevalence of malnutrition indicators across IDD subtypes.

Variables^a^	Children with ASD (*n* = 21)	Children with DS (*n* = 10)	Children with ADHD (*n* = 16)	Children with CP (*n* = 21)	*χ* ^2^ (df)	*p*	*q* (FDR)	Cramer's *V*
Underweight^b^								
Yes	0 (0%)	0 (0%)	3 (30%)	2 (17%)	18.44	0.135	0.002	0.52
No	13 (100%)	5 (100%)	7 (70%)	10 (83%)	(3)			
Severe underweight^b^								
Yes	0 (0%)	0 (0%)	2 (20%)	0 (0%)	10.82	0.097	0.031	0.40
No	13 (100%)	5 (100%)	8 (80%)	12 (100%)	(3)			
Stunting								
Yes	0 (0%)	5 (50%)	6 (38%)	1 (5%)	4.68	**0.001**	0.236	0.26
No	21 (100%)	5 (50%)	10 (63%)	20 (95%)	(3)			
Severe stunting								
Yes	0 (0%)	2 (20%)	4 (25%)	0 (0%)	7.49	**0.013**	0.116	0.33
No	21 (100%)	8 (80%)	12 (75%)	21 (100%)	(3)			
Thinness								
Yes	0 (0%)	2 (20%)	3 (19%)	2 (10%)	20.15	0.197	0.002	0.54
No	21 (100%)	8 (80%)	13 (81%)	19 (91%)	(3)			
Severe thinness								
Yes	0 (0%)	2 (20%)	2 (13%)	0 (0%)	17.17	0.058	0.003	0.50
No	21 (100%)	8 (80%)	14 (88%)	21 (100%)	(3)			
Undernutrition								
Yes	0 (0%)	6 (60%)	8 (50%)	3 (14%)	3.39	**0.001**	0.366	0.22
No	21 (100%)	4 (40%)	8 (50%)	18 (86%)	(3)			
Severe undernutrition								
Yes	0 (0%)	4 (40%)	5 (31%)	0 (0%)	12.02	**0.001**	0.022	0.42
No	21 (100%)	6 (60%)	11 (69%)	21 (100%)	(3)			
Overweight								
Yes	2 (10%)	1 (10%)	2 (13%)	5 (24%)	5.56	0.557	0.180	0.37
No	19 (91%)	9 (90%)	14 (88%)	16 (76%)	(3)			
Obese								
Yes	3 (14%)	4 (40%)	1 (6%)	2 (10%)	6.32	0.091	0.146	0.40
No	18 (86%)	6 (60%)	15 (94%)	19 (91%)	(3)			
Overnutrition								
Yes	5 (24%)	5 (50%)	3 (19%)	7 (33%)	2.08	0.335	0.557	0.17
No	16 (76%)	5 (50%)	13 (81%)	14 (67%)	(3)			
Malnutrition								
Yes	5 (24%)	9 (90%)	8 (50%)	10 (48%)	6.47	**0.007**	0.146	0.31
No	16 (76%)	1 (10%)	8 (50%)	11 (52%)	(3)			

^a^ Categorical variables were analyzed by using chi‐squared test and expressed as numbers and percentages. Significant values (*p* < 0.05) were presented in bold type.

^b^ Children with IDD (≤ 10 years); *n* = 40 (ASD *n* = 13; DS *n* = 5; ADHD *n* = 10; CP *n* = 12).

**FIGURE 1:**
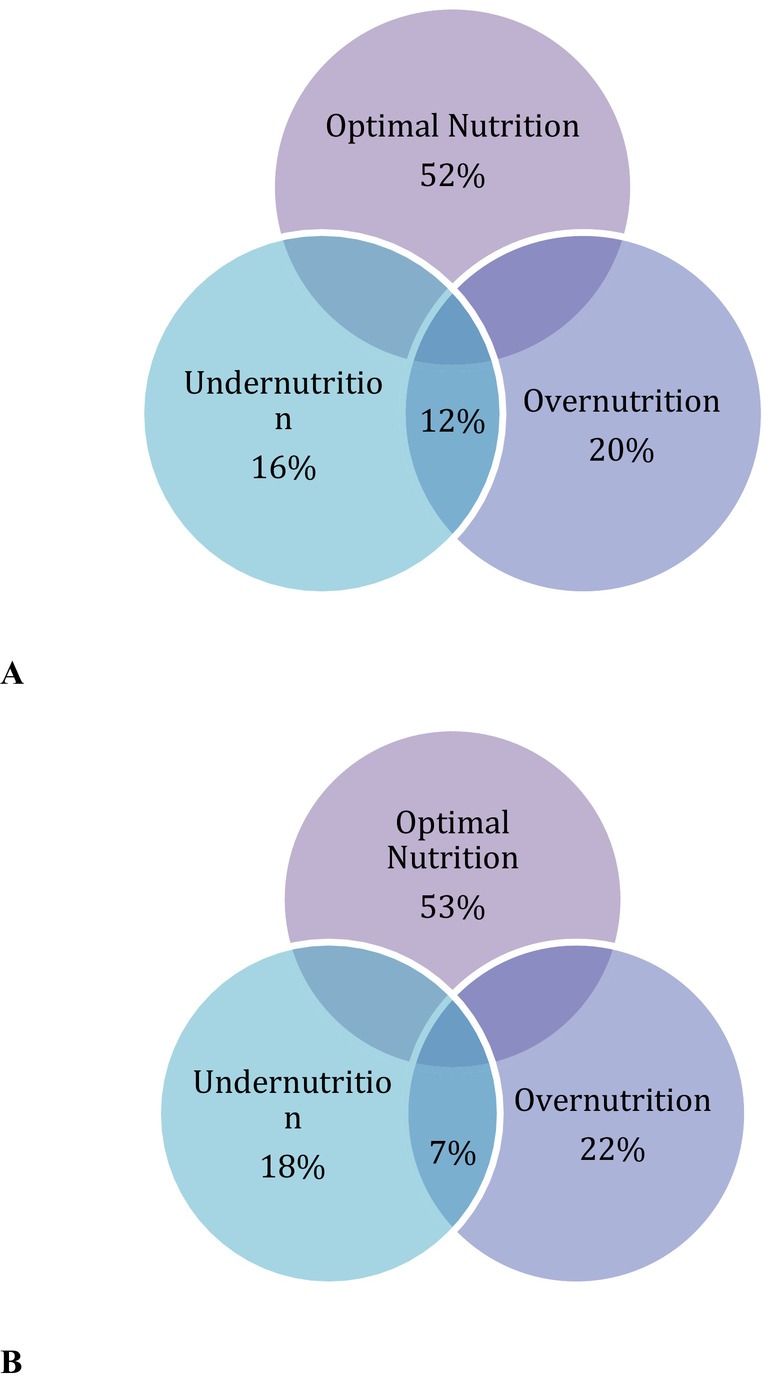
Venn diagram showing relationship between various forms of malnutrition (undernutrition and overnutrition) in (A) healthy participants, and (B) participants with IDD.

Several children exhibited the double burden of malnutrition, as illustrated in Figure 1. Among healthy participants, 12 children (12%) were found to have overnutrition (overweight *n* = 2, obesity *n* = 3), as well as undernutrition (all of them had stunting, and four children were severely stunted). Similarly, among participants with IDD, five children (7%) were found to have overnutrition (overweight, *n* = 4; obesity, *n* = 8), as well as undernutrition (all of them had severe stunting).

## 4 Discussion

To the best of our knowledge, this is the first study to investigate the prevalence of malnutrition among school‐aged children with and without IDD in Saudi Arabia. Our findings suggest that malnutrition was substantially prevalent in the children in this study. Our data show a higher prevalence of malnutrition in the form of overnutrition than in the form of undernutrition. To assess different forms of malnutrition among the child population, WHO standards, including BAZ, HAZ, and WAZ, are designed for growth assessment among school‐aged children and adolescents (5–19 years) (De Onis et al. 2007). Unfortunately, global estimates for malnutrition among this population, either with or without IDD, remain sparse compared with those among preschool children aged under 5 years (NCD RisC 2024). Results obtained by pooling data from 222 million participants in 200 countries revealed that from 1990 to 2022, the combined prevalence of thinness and obesity increased in most countries, mainly due to increased obesity prevalence. By 2022, obesity prevalence exceeded thinness prevalence for both boys and girls aged 5–19 years in most countries around the world (NCD RisC 2024). A recent systematic review and meta‐analysis covering more than 45 million participants assessed the global prevalences for overweight and obesity at 14.8% and 8.5%, respectively, among children and adolescents less than 18 years, with significant cross‐country variation (Zhang et al. 2024). For adolescents, thinness has slightly decreased since 2010 (about 12% in boys and 8% in girls aged 10–19 years), but it has substantially increased in several regions. Comparable global rates for malnutrition in children aged 5–17 years are less consistently reported, although many low‐ and middle‐income countries continue to record high rates of stunting and thinness (Wrottesley et al. 2023).

According to UNICEF's regional study in the Middle East and North Africa (MENA) in 2024, 33% of school‐aged children and adolescents (5–19 years old) were overweight or obese, while 6.9% were thin. At least one‐fifth of school‐aged children and adolescents (5–19 years) were overweight or obese in all but two countries. Countries with the highest prevalences include Kuwait and Qatar (47%), the United Arab Emirates (45%), Egypt and Lebanon (42%), and Bahrain and Saudi Arabia (41%) (UNICEF MENA 2024). However, adolescent stunting and wasting estimates are not comparable across the region, while data on children under 5 years are more comprehensive (UNICEF MENA 2024).

Nearly half of the participants with and without IDD had at least one form of malnutrition, and overnutrition exceeded undernutrition. While overall malnutrition prevalence was similar between groups, the pattern within the IDD group was heterogeneous and clinically relevant, with DS and ADHD showing particularly high burdens. International evidence often shows higher undernutrition risk among children with disabilities than among children without disabilities. For example, multi‐country analyses have reported higher prevalence ratios for stunting, wasting, and underweight in children with disabilities (Rotenberg et al. 2024). In our study, the absence of an overall difference may reflect several factors: (1) the comparison group was hospital‐based rather than community‐based, which can inflate malnutrition prevalence in children without IDD; (2) clinic‐based recruitment of children with IDD may capture families receiving ongoing care and nutrition counseling; and (3) limited statistical power to detect modest differences (Type II error).

Within IDD subtypes, DS showed the highest overall malnutrition prevalence, driven by both high obesity and high stunting. This reinforces the need for integrated clinical follow‐up that addresses both excess adiposity and linear growth impairment, including screening for endocrine comorbidities, individualized dietetic counseling, and physical activity promotion adapted to ability (Bertapelli et al. 2016; Basil et al. 2016). Children with ADHD also showed a high burden of undernutrition and severe undernutrition; appetite effects of stimulant medications and irregular meal patterns may contribute and warrant growth monitoring (Cortese et al. 2016; Zhu et al. 2024). Children with CP commonly experience feeding and swallowing difficulties, underscoring the importance of feeding safety and individualized nutritional support (Rebelo et al. 2022; Almuneef et al. 2019).

The observed individual‐level double burden, concurrent stunting with overweight/obesity, highlights that nutrition risks do not occur in isolation. This pattern has been increasingly reported globally, including in middle‐ and high‐income settings, and suggests that interventions must address diet quality and growth monitoring rather than focusing exclusively on either obesity prevention or undernutrition treatment (Viana et al. 2025; Katoch and Nawaz 2025).

Even though existing data from Saudi Arabia about nutritional status in children with and without IDD are scarce, the limited number of available studies show that both forms of malnutrition (undernutrition and overnutrition) were substantially prevalent (AlFaris et al. 2024). A nationwide representative study found moderate stunting (about 11%) and severe stunting (about 2%) among healthy Saudi children and adolescents aged 5–17 years (El Mouzan et al. 2011). At the same time, Saudi Arabia was ranked among countries with very high overweight and obesity rates in school‐aged children and adolescents (41%), consistent with the nutritional transition observed in the Saudi community (UNICEF MENA 2024). Data from Saudi Arabia on children aged 2–18 years also highlight the coexistence of undernutrition and overnutrition, with a study reporting moderate underweight (4.7%, severe was 1.5%), moderate wasting (6.3%, severe was 2.6%), alongside overweight (20.8%) and obesity (11.3%) (Alshammari et al. 2017). Together, these findings suggest a substantial prevalence of several forms of malnutrition in the childhood population in Saudi Arabia.

For all children, particularly those with IDD, regular growth monitoring using WHO school‐age standards, early screening for feeding problems, and relevant nutrition care, including dietetic management and physical activity promotion adapted to ability, are needed to reduce both undernutrition and overnutrition (Wong and Srivastava 2023; Maiano et al. 2016; Zablotsky et al. 2019). At the population level, recent global trends suggest that obesity is now exceeding thinness in most countries, while thinness persists in specific regions around the world. Saudi data indicate both persistent stunting rates and high overweight/obesity rates in school‐aged children (Alman et al. 2021; Grondhuis and Aman 2014). Evidence from Riyadh also points to challenges in school food environments and only moderate compliance with school canteen nutrition policies, suggesting the need for stronger implementation and monitoring (Aldubayan and Murimi 2019). These findings support the need for integrated policies that combine healthy food environments, such as limits on ultra‐processed foods and sugary drinks in/around schools, with high‐quality nutrition care that manages the specific barriers facing children with and without IDD (Cardel et al. 2020; Jebeile et al. 2022; Must et al. 2014).

Our findings support a dual strategy for Saudi Arabia: (1) strengthening school‐based nutrition environments (e.g., healthier canteen offerings, restrictions on sugar‐sweetened beverages, and implementation monitoring) and (2) integrating routine nutrition screening into pediatric and developmental clinics with clear referral pathways to dietitians and feeding specialists (Aldubayan and Murimi 2019; Must et al. 2014). For children with IDD, programs should be tailored: DS‐focused protocols may prioritize obesity prevention while monitoring linear growth (Bertapelli et al. 2016); CP‐focused care should emphasize feeding safety and adequate energy/protein intake (Rebelo et al. 2022); and ADHD‐focused follow‐up should include appetite and growth monitoring during pharmacotherapy (Cortese et al. 2016). At the national level, aligning child health services with regional strategies that aim to halt the rise in childhood overweight and improve diet quality can help address the double burden of malnutrition (UNICEF MENA 2024).

This study has limitations. First, recruitment was single‐center and clinic‐based, which limits generalizability and introduces selection bias. Second, the comparison group was hospital‐attending rather than community‐based, which may overestimate malnutrition prevalence among children without IDD. Third, group sizes were imbalanced (non‐IDD *n* = 100 vs. IDD *n* = 68), and IDD subtype samples were modest, limiting power and increasing the risk of Type II error. Fourth, micronutrient status, physical activity, and pubertal stage were not assessed, and disability severity was not examined due to limited numbers. Nevertheless, this study still provides an exploratory overview on an important field to inform tailored interventions and a call for future research with representative sample sizes and more solid methodologies.

## 5 Conclusion

In summary, school‐aged children with and without IDD were found to have a high prevalence of malnutrition, with the prevalence of overnutrition exceeding that of undernutrition. These findings underscore the need for fundamental nutritional screening, periodic nutritional assessments, and appropriate nutritional care of children with and without IDD. Such monitoring and care are crucial to managing and preventing malnutrition among children with and without IDD in both clinical and school settings. Further research with representative samples and different age groups of children with and without IDD is required to acquire a deeper understanding of malnutrition prevalence, related determinants, and appropriate prevention and management strategies.

## Author Contributions

**Jozaa Z. ALTamimi:** conceptualization, investigation, writing – original draft, visualization, supervision. **Nora A. AlFaris:** conceptualization, validation, data curation, supervision, writing – original draft. **Naseem M. Alshwaiyat:** methodology, software, formal analysis, data curation, writing – original draft. **Lujain A. Almousa:** conceptualization, resources, writing – original draft, project administration, funding acquisition. **Malak A. Alsemari:** methodology, software, validation, visualization, writing – review and editing. **Reham I. Alagal:** methodology, formal analysis, investigation, project administration, writing – original draft.

## Funding

This work was supported by Princess Nourah bint Abdulrahman University (PNURSP2026R129).

## Conflicts of Interest

The authors declare no conflicts of interest.

## Data Availability

Data will be available from the corresponding author on reasonable request.
